# CD3 and PD-L1 tissue expression have synergistic value in head and neck squamous cell carcinoma prognosis

**DOI:** 10.1016/j.tranon.2026.102776

**Published:** 2026-04-16

**Authors:** Adrian v. Witzleben, Romain Remark, Christian Idel, Julika Ribbat-Idel, Rosemarie Krupar, Andreas Schröck, Niklas Klümper, Johannes Doescher, Andrew G. Sikora, Tsima Abou Kors, Julius M Vahl, Matthias Brand, Michael Sonntag, Cornelia Brunner, Thomas K Hoffmann, Sven Perner, Sacha Gnjatic, Simon Laban

**Affiliations:** a Department of Otorhinolaryngology and Head & Neck Surgery, Ulm University Medical Center, Ulm, Germany; b Department of Immunology and Immunotherapy, Icahn School of Medicine at Mount Sinai, Mount Sinai Hospital, New York City, NY, USA; c Department of Otorhinolaryngology and Head & Neck Surgery, Universitätsklinikum Schleswig Holstein, Campus Lübeck, Germany; d Institute of Pathology, Universitätsklinikum Schleswig Holstein, Campus Lübeck, Germany; e Department of Otorhinolaryngology, Universitätsklinik Bonn, Germany; f Department of Urology, Universitätsklinik Bonn, Germany; g Department of Otolaryngology, Augsburg University Hospital, Augsburg, Germany; h Department of Head and Neck Surgery, Division of Surgery, MD Anderson Cancer Center, USA; i PATHORA Institut für Pathologie und Gewebsmedizin, Tübingen, Reutlingen, Germany

**Keywords:** HNSCC, Tumor microenvironment, T cells, CD3, PD-L1

## Abstract

**Introduction:**

T cell infiltrates, particularly CD3^+^ T cells, have been linked to a favorable prognosis in cancer. However, the influence of PD-L1 expression on survival remains controversial, particularly in patients who do not receive immune checkpoint inhibition. This goal of this study was to combine CD3 density and PD-L1 expression to assess their individual and combined prognostic value in head and neck squamous cell carcinoma (HNSCC) patients treated with surgery and adjuvant therapy.

**Materials and Methods:**

A tissue microarray of 458 HNSCC primary tumor samples was analyzed for CD3 and PD-L1 expression using multiplex immunohistochemistry. CD3 densities were quantified using digital image analysis (QuPath), and PD-L1 expression was categorized by the Combined Positive Score (CPS). Overall survival (OS) and recurrence-free survival (RFS) were calculated and compared across different expression profiles using the Kaplan-Meier method followed by a multivariate cox regression analysis.

**Results:**

CD3^high^ tumors (defined as greater the median CD3 expression) were associated with longer OS and RFS compared to CD3^low^ tumors (below median). Similarly, PD-L1 expression at CPS ≥ 1 was linked to significantly better survival outcomes than CPS < 1. The combination of CD3^high^ and PD-L1 CPS ≥ 1 showed the most favorable prognosis.

**Conclusion:**

The combined assessment of CD3 density and PD-L1 expression outperforms either marker alone in predicting survival outcomes in surgically treated patients without immunotherapy. CD3^high^ and PD-L1 CPS ≥ 1 identifies patients with favorable prognosis and response to standard-of-care treatment. Nonetheless, the potential relevance of these markers for guiding immunotherapy in other setting remains to be explored.

## Highlights

• CD3^high^ and PD-L1 CPS ≥ 1 each predict better OS and RFS in HNSCC.
• Best outcomes seen in patients with both CD3^high^ and PD-L1 CPS ≥ 1.
• Combined markers outperform either marker alone for prognosis.
• Markers may help identify patients with favorable prognosis under standard care.
• Predictive value for immunotherapy remains to be clarified.

## Introduction

Head and neck squamous cell carcinoma (HNSCC) is a common cancer type, with nearly 900,000 new cases and approximately 450,000 deaths reported annually [1]. Approximately 25% of all HNSCC cases and up to 60% of oropharyngeal squamous cell carcinomas (OPSCC) are associated with human papillomavirus (HPV) [2]. Chronic infection with a high-risk HPV subtype can lead to OPSCC development, independent of traditional risk factors such as smoking and alcohol consumption [3]. The distinct carcinogenesis pathways of HPV^pos^ and HPV^neg^ HNSCC result in unique (immune-)molecular phenotypes [[4], [5], [6], [7], [8], [9]]. Most HPV^pos^ OPSCC patients exhibit high levels of tumor-infiltrating lymphocytes (TIL) in their tumor tissue, partly comprised of HPV-specific immune cells, as a response to viral antigens [7,[10], [11], [12], [13], [14], [15]]. Similarly, in carcinogen-driven HNSCC, high immune cell infiltration is associated with improved overall and recurrence-free survival [16]. Interestingly, the majority of infiltrating immune cells are CD8^+^ T cells, followed by CD4^+^ T cells [17]. However, anti-tumor immune cells are primarily suppressed by regulatory T cells and, in certain contexts, also by Th17 cells, which inhibit the activity of CD8^+^ and CD4^+^ T cells [18].

It has been demonstrated that an inflamed tumor microenvironment in HNSCC is associated with improved response rates to anti-PD1 treatment [13]. Evaluating the expression of the co-inhibitory molecule PD-L1 via immunohistology is essential for clinical decision-making regarding the immunotherapy of these patients. However, the prognostic role of PD-L1 expression in HNSCC remains controversial. Some studies have reported a worse prognosis for HNSCC patients with high PD-L1 expression [[19], [20], [21], [22]]. Other groups have found no significant differences [23,24], while some analyses suggest a better prognosis associated with high PD-L1 expression [25,26]. Recently, neoadjuvant and adjuvant pembrolizumab has been approved for patients with locoregionally advanced HNSCC and a CPS ≥ 1 by FDA based on the phase 3 study Keynote-689 [27].

Therefore, a more comprehensive prognostic evaluation needs to be set up with the involvement of immune cell densities and PD-L1 expression at the same time, especially in the era of straightforward assays for multiplex immunohistochemistry.

As there are data showing significant differences among anatomical subsites in HNSCC regarding the immune cell densities [28]. Here, we determined the prognostic impact of T cell densities and PD-L1 expression in HNSCC patients undergoing surgery and adjuvant therapy.

## Material and methods

### Patient cohort and sample collection

Between 1997 and 2011, patients received treatment in accordance with local clinical guidelines at the University Hospital of Bonn, Germany. After completion of clinical diagnostics, archived formalin-fixed paraffin-embedded tissue samples were used to construct a tissue microarray (TMA). Representative 0.6 mm cores were taken from the samples and integrated into TMA blocks. The TMA encompassed 458 primary tumors, with each tumor specimen represented in triplicate. The study received approval from the University Hospital of Bonn's internal review board (reference #174/13). Details on the impact of known prognostic factors in the cohort have been published previously [29]. Patient characteristics are shown in Table 1.

**Table 1:** Patient characteristics.

Primary Site		Oral Cavity n = 116		Oropharynx n = 137		Hypopharynx n = 54		Larynx n = 151		Total n = 458	
Age at diagnosis (years, mean)		64y		59y		61y		64y		62y	
Gender	male	66	57%	102	74%	46	85%	129	85%	343	74.9%
	female	50	43%	35	26%	8	15%	22	15%	115	25.1%
T Status	T1/2	95	82%	84	61%	27	50%	114	75%	320	69.7%
	T3/4	20	17%	53	38%	27	50%	37	25%	137	29.8%
	na	1	1%	1	1%					2	0.4%
N Status	N0	68	59%	36	26%	14	26%	128	85%	246	53.8%
	N+	47	41%	99	73%	40	74%	23	15%	209	45.7%
	na	1	1%	1	1%					2	0.4%
HPV-status (DNA + p16)	negative	109	95%	116	85%	52	96%	151	100%	428	93.7%
	positive	5	4%	20	15%	2	4%	0	0%	27	5.9%
	na	1	1%	1	1%					2	0.4%
Smoking status	non-smoker	12	10%	21	15%	3	6%	7	5%	43	9.4%
	smoker	56	48%	92	67%	43	80%	119	79%	310	67.7%
	na	48	41%	24	18%	8	15%	25	17%	105	22.9%

HPV-status was defined by the results of HPV-PCR and P16 immunohistochemistry. Only HPV-DNA+ and p16+ patients were considered HPV-positive, whereas all other combinations were considered HPV-negative.

### Multiplex immunohistochemistry

The slides were stained immunohistochemically for CD3 and PD-L1 on the same slides in a stain and strip procedure named MICSSS (multiplexed immunohistochemical consecutive staining on single slide) as previously published with a different selection of markers [30,31]. Anti-CD3 staining was performed with prediluted rabbit IgG clone 2GV6 after antigen retrieval at pH 9, and PD-L1 staining used rabbit IgG clone E1L3N at pH9 and 1/100 dilution. The stained TMA slides were digitized using the Zeiss MIRAX DESK scanner. Then immune cell (IC) densities per mm² were quantified using the open-source digital image analysis software (QuPath), and individual means were calculated from triplicate samples. PD-L1 expression was assessed using the Combined Positive Score as previously described from triplicates on the TMA(CPS)[32].

### Statistics

Overall Survival (OS) was defined as the time from the date of diagnosis until death or latest follow-up. Recurrence-free survival (RFS) was defined as the time from the date of diagnosis until recurrence or death (whichever occurred first) or latest follow-up. Survival times were calculated using the Kaplan-Meier method and compared by log-rank tests in IBM SPSS (Version 29). IC infiltrates from different primary tumor sites were compared using Kruskal-Wallis and Mann-Whitney *U* tests, with paired samples analyzed using the Wilcoxon signed-rank test. CD3 IC densities were classified as high or low based on the individual median for each primary tumor site (suppl. Fig. 1).

PD-L1 CPS scores were scored into three categories CPS < 1 and CPS ≥ 1 / CPS <20 or CPS ≥ 20 (suppl. Fig. 3).

The multivariate Cox regression analysis was performed using SPSS version 29 using the backward stepwise method and a likelihood ratio including CD3/PD-L1 (cold vs. hot), HPV-status (neg vs. pos), N-status (N0 vs*.* N+) and T-status (T1/2 *vs*. T3/4).

Fig.s were graphed using SPSS (Version 29) and GraphPad Prism (Version 10).

## Results

### Patient characteristics

**Fig. 1:**
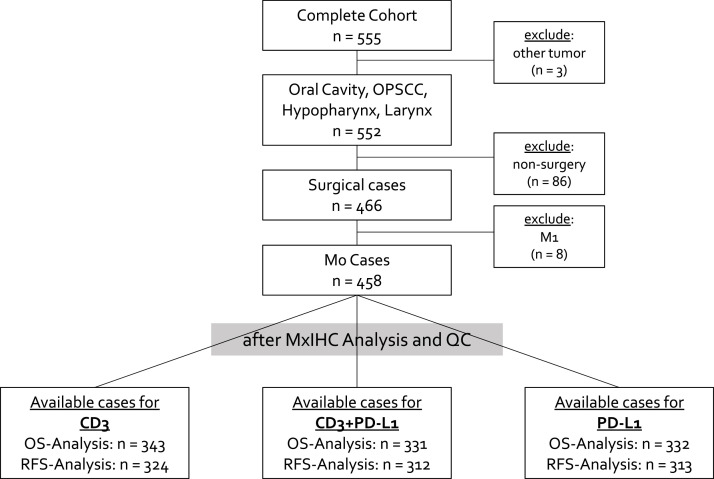
Consort Diagram displaying the selection and available samples of our HNSCC cohort for the study.

The HNSCC patient cohort included 458 primary tumor cases representing patients with oral cavity, oropharynx, larynx, and hypopharynx cancer which were treated surgically without signs of distant metastasis. For each analysis step, cases with missing data for the respective analysis were excluded. A consort diagram is shown in Fig. 1 for the detailed selection and availability of the analyzed patient cases after primary TMA analysis and quality control.

Detailed patient characteristics by primary tumor site are presented in Table 1.

### CD3 and PD-L1 function as independent prognostic markers

**Fig. 2:**
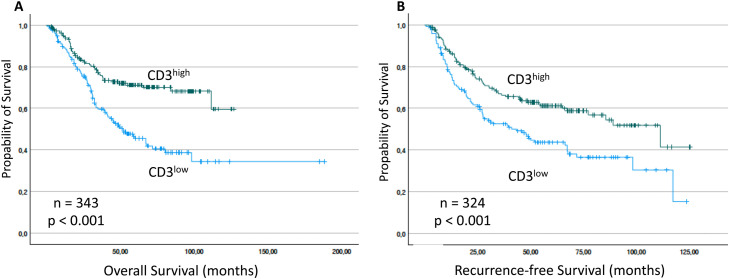
Overall survival (OS) and Recurrence-free survival (RFS)of patients stratified by CD3 expression. OS and RFS (months) was significantly reduced for patients with low CD3 expression. The *p*-values for pairwise comparisons are indicated.

In order to validate the prognostic impact of CD3 expression on OS and RFS in surgically treated patients (see consort diagram in Fig. 1) we performed survival analyses using the Kaplan-Meier method. Cases were classified as CD3^high^ or CD3^low^ based on the CD3 density compared to the median CD3 density of the respective primary tumor site (suppl. Fig. 1; example image: suppl. Fig. 2). OS was significantly lower for patients showing a low density of CD3 (CD3^low^: mean OS = 88.7 months vs. CD3^high^: mean OS = 94.3, *p* < 0.001; *n* = 343 / Fig. 2A). Similarly, RFS was significantly lower for patients showing a low CD3infiltration (CD3^low^: mean RFS = 58.4 months vs. CD3^high^: mean RFS = 79.2, *p* < 0.001; *n* = 324 / Fig. 2B).

**Fig. 3:**
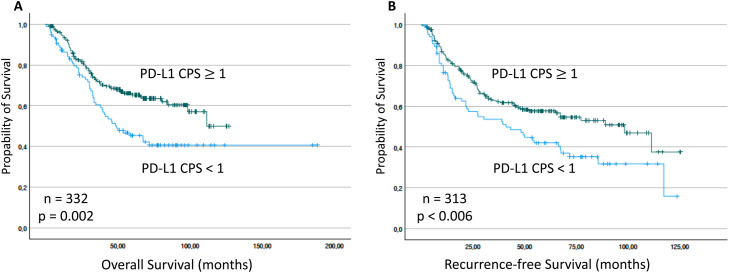
Overall survival (OS) and Recurrence-free survival (RFS) of patients stratified by PD-L1 (CPS) expression. OS and RFS (months) was significantly reduced for patients expressing with PD-L1 CPS <1. The *p*-values for pairwise comparisons are indicated.

For PD-L1 expression, first three groups (CPS < 1, CPS 1 - 19 and CPS ≥ 20) were compared. for the prognostic impact of CPS 1 - 19 and CPS ≥ 20 was similar (suppl. Fig. 3) Therefore PD-L1 expression was dichotomized using the CPS value of 1 (CPS < 1 and CPS ≥ 1). Here OS was significantly shorter for patients with a CPS < 1 (PD-L1 CPS < 1: mean OS = 94.2 months vs. PD-L1 CPS ≥ 1: mean OS = n.a., *p* = 0.002; *n* = 332 / Fig. 3A). Also, RFS was significantly shorter for patients showing a low CPS (PD-L1 CPS < 1: mean RFS = 57.2 months vs. PD-L1 CPS ≥ 1: mean RFS = 75.1, *p* < 0.006; *n* = 313 / Fig. 3B).

### CD3 and PD-L1 co-staining shows superior prognostic value for both OS and RFS

**Fig. 4:**
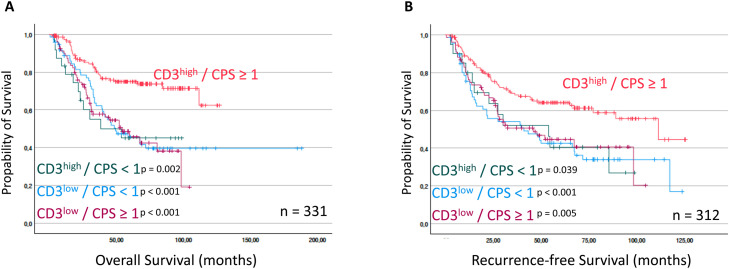
Overall survival (OS) and Recurrence-free survival (RFS) of patients stratified by combined CD3 and PD-L1 expression. OS and RFS (months) was significantly reduced for patients expressing a high CD3 and a CPS ≥1. The *p*-values for comparisons are indicated.

The combined assessment of CD3 density and PD-L1 expression provides improved prognostic accuracy compared to either marker alone. The co-expression of CD3^high^ and PD-L1 CPS ≥ 1 was associated with the best survival outcomes – both OS and RFS – while the other three combinations CD3^low^ CPS < 1, CD3^low^ CPS ≥ 1 and CD3^high^ CPS < 1 showed comparably worse survival rates (OS and RFS: Fig. 4A and B). Here the OS was significantly better for patients showing a “hot” combination of CD3^high^ and PD-L1 CPS ≥ 1 in comparison to the other “cold” combinations (“hot”: mean OS = 98.17 months, *p* < 0.001 *vs*. “cold”: mean OS = 89.62, *p* = 0.002; *n* = 331 / Fig. 4A). Also, the RFS was significantly higher for patients showing a “hot” immune infiltrate of CD3^high^ and PD-L1 CPS ≥ 1 (“hot”: mean RFS = 82.10 months vs. “cold”: mean RFS = 58.02 *p* = 0.002; *n* = 312 / Fig. 4B). A combined Kaplan-Meier curve with dichotomized groups of “hot” vs. “cold” is shown in suppl. Fig. 4.

### CD3/PD-L1 is a strong and independent marker of OS and RFS in HNSCC

A multivariate cox regression (backward stepwise, likelihood ratio) analysis was performed using known prognostic factors (HPV-status: negative vs. positive, N category (N0 vs. N+), and T category (T1/2 vs. T3/4)) and dichotomized CD3/PD-L1 status (CD3^high^ PD-L1 CPS ≥ 1 = “hot”; CD3^low^ CPS < 1, CD3^low^ CPS ≥ 1 and CD3^high^ CPS < 1 = “cold”).

**Fig. 5:**
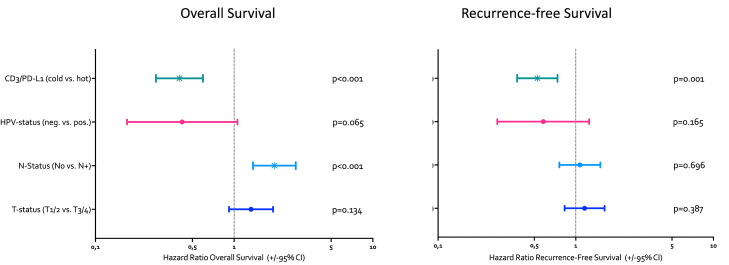
Hazard ratio for overall survival (OS) and Recurrence-free survival (RFS) of patients stratified by combined CD3 and PD-L1 expression (cold vs. hot), HPV (human papilloma virus) status Nodal (N)-Status and Tumor (T)-Status. The *p*-values for the multivariate testing are indicated*. CI = confidence interval*.

Hazard ratios (HR) with 95% confidence intervals and p-values of the multivariate analysis for OS and RFS are shown in Fig. 5. For OS “hot” CD3/PD-L1 status (HR = 0.422, *p* < 0.001) and N-status (HR = 1.951, *p* < 0.001) were confirmed as independent prognostic factors. HPV-status (HR = 0.422, *p* = 0.065) and T-status (HR = 1.323, *p* = 0.134) did not reach statistical significance.

For RFS, only “hot” CD3/PD-L1 status (HR = 0.526, *p* < 0.001) was confirmed as an independent prognostic factor. HPV-status (HR = 0.580, *p* = 0.165) N-status (HR = 1.071, *p* = 0.696) and T-status (HR = 1.159, *p* = 0.387) did not reach statistical significance.

## Discussion

Future cancer therapy relies on reliable prognostic biomarkers to optimize treatment strategies and improve patient outcomes, particularly in the era of immunotherapy and personalized medicine. In this study, we identified an improved prognostic value of combining CD3⁺ T-cell density with PD-L1 status compared with using either marker alone. Notably, one intriguing finding is the association between PD-L1 positivity and improved survival in a cohort treated with standard-of-care surgery without immune checkpoint inhibition. While PD-L1 expression is commonly viewed as a mechanism of immune suppression and tumor immune escape, our results support a context-dependent interpretation in which PD-L1 may primarily reflect a pre-existing, active anti-tumor immune response rather than an intrinsically adverse biological feature.

Recently, neoadjuvant and adjuvant pembrolizumab has been approved for patients with locoregionally advanced HNSCC who are candidates for surgery with a CPS ≥ 1 based on the phase III trial Keynote-689 [33]. Thus, predictive markers for treatment response are needed for locoregionally advanced disease.

While PD-L1 expression has been widely studied as a prognostic and predictive marker in various cancers and HNSCC, its standalone prognostic value remains limited. In our study we found a significant survival benefit for HNSCC patients with a combined positivity score (CPS) ≥ 1 similarly to the published studies from Balermpas et al. und Lecerf et al. [25,26]. PD-L1 expression (CPS/tumor proportion score: TPS) has been established for HNSCC as a biomarker for pembrolizumab treatment of recurrent/metastatic HNSCC in Europe.

We hypothesized that adding CD3 density to PD-L1 expression may improve prognostic accuracy.

Firstly, we addressed anatomically determined differences in the densities of CD3 infiltrates by primary site. Our analysis of CD3 densities across the different subsites of HNSCC confirmed previously published differences in immune cell densities [28]. Thus, we used a site-specific cut-off to define CD3^high^ and CD3^low^.

Combining prognostic markers can increase prognostic discrimination. An example is the analysis of tissue-resident memory T cells (T_RM_) using combined staining for CD8 and CD103, which represents the key cell population for the adaptive anti-tumor immune response. This is also reflected in the superior survival of patients with high T_RM_ densities and their better response to immunotherapy [34,35].

An important limitation of the present analysis is that CD3 immunohistochemistry does not allow discrimination between functionally distinct T-cell subsets, such as cytotoxic CD8⁺ T cells, CD4⁺ helper T cells, or regulatory T cells. Consequently, a high CD3⁺ T-cell density may not exclusively reflect effective antitumor immunity but could also indicate the presence of immunosuppressive T-cell populations or a cytokine milieu that counteracts antitumoral responses. This hampers a more refined functional interpretation of CD3 as a prognostic marker. Future studies incorporating subset-specific markers and functional characterization of the tumor immune microenvironment will be essential to disentangle these effects and to better define the prognostic and biological relevance of intratumoral T-cell infiltration.

Another limitation of our study is the lack of assessment of myeloid immune populations, such as tumor-associated macrophages (TAMs), neutrophils, and monocytes, which can influence both PD-L1 expression and patient outcomes. In multiple solid tumor models, infiltration by TAMs correlates with elevated PD-L1 expression on tumor cells, suggesting that myeloid cells can act as extrinsic regulators of PD-L1 beyond lymphocyte-driven interferon-γ signaling. For example, increased TAM infiltration is associated with PD-L1 positivity in lung adenocarcinoma and can upregulate PD-L1 on tumor cells via macrophage-derived factors in vitro and in vivo, independently of T-cell density [36]. Similarly, M2-polarized TAMs have been shown to enhance PD-L1 expression in cervical carcinoma through PI3K/AKT signaling and correlate with poorer prognosis [37]. Moreover, tumor-resident macrophages exhibit higher PD-L1 than circulating monocytes, indicating local microenvironmental regulation of checkpoint expression on myeloid cells themselves [38]. The omission of myeloid profiling therefore limits a full understanding of the immunosuppressive microenvironment and its impact on PD-L1 as a prognostic marker. Future studies integrating both lymphoid and myeloid immune cell characterization will help clarify how these distinct populations jointly influence immune escape mechanisms and patient outcomes.

Our findings demonstrate that the combined simple assessment of CD3 density and PD-L1 expression significantly improves prognostic precision. Patients with high CD3 infiltration and a PD-L1 CPS value ≥1 exhibited the most favorable overall and relapse-free survival outcomes compared to all other three combinations. This reinforces our initial hypothesis that, on the one hand, an active immune microenvironment (expressing PD-L1 as an immune modulating exhaustion and activation marker) and, on the other hand, a high T cell infiltration contributes to better tumor control in SOC surgically treated patients. In fact, tumors with CD3^low^ T cell density and CPS ≥ 1, and CD3^high^ patients with CPS ≤ 1 displayed comparably poor survival outcomes as patients with both CD3^low^ and CPS ≤ 1. Hence, the quantity of TILs alone does not lead to better survival - the quality and specific cell type of TILs and their activation status seem to be important. Especially with regards to immunotherapy [39]. These results suggest that integrating CD3 and PD-L1 expression as a dual biomarker strategy may refine prognostic classifications and inform clinical decision-making- especially in the context of neoadjuvant and adjuvant pembrolizumab.

The combination of CD3 and PD-L1 outperformed clinically established prognostic markers like T-, N- and HPV-status.

The low prevalence of HPV-positive patients in the cohort may have resulted in an underestimation of the prognostic impact of HPV-status. But in the Keynote-689-trial, the rate of HPV-positive patients was also comparably low [33].

The use of multiplex immunohistochemistry (IHC) represents a major advancement in biomarker evaluation, enabling the simultaneous assessment of multiple markers in a single tissue section and offering comprehensive insights into the tumor immune landscape [30,[40], [41], [42], [43], [44], [45], [46], [47], [48]]. Its integration into routine diagnostics may enhance personalized treatment decision strategies through precise patient stratification and tailored interventions. A key challenge in prognostic biomarker research is identifying markers that are both biologically relevant and practical for clinical use—ideally, they should be easily measurable, cost-effective, and reproducible in standard pathology laboratories. Combining multiple markers with well-established assays has emerged as a promising strategy to improve prognostic accuracy while maintaining clinical feasibility like CD3 and PD-L1. An additional aspect relevant to the interpretation of our results is the cellular source of PD-L1 expression. The combined positive score (CPS) integrates PD-L1 expression on both tumor cells and infiltrating immune cells, precluding a compartment-specific analysis in the present study. However, immune-cell PD-L1 expression has been shown to be particularly enriched in inflamed tumors and may reflect ongoing immune activation rather than tumor-intrinsic immune escape. In this context, CPS-based PD-L1 positivity may preferentially identify tumors with a pre-existing adaptive immune response, especially when accompanied by high CD3⁺ T-cell densities. Future studies incorporating spatially resolved or multiplexed analyses will be required to disentangle the relative contributions of tumor-cell versus immune-cell PD-L1 to prognosis.

While our findings provide strong evidence for the prognostic utility of CD3 and PD-L1 co-evaluation, further research is necessary to validate these observations in the context of neoadjuvant and adjuvant pembrolizumab to confirm that “hot” CD3/PD-L1 can be used to predict treatment benefit. Expanding these studies also across different cancer types and treatment settings such as immunotherapy response, will be crucial for confirming the broader applicability of this biomarker combination.

In conclusion, integrating CD3 and PD-L1 expression as prognostic biomarkers represents a significant step toward determining the patient’s prognosis and in future personalized cancer therapy. This approach not only enhances the accuracy of survival predictions but also contributes to a more comprehensive understanding of tumor-immune interactions. By incorporating these biomarkers into routine diagnostics, clinicians may be able to make more informed treatment decisions, ultimately improving patient outcomes and optimizing cancer care.

## Disclosures

Sacha Gnjatic reports research funding from Boehringer Ingelheim, Bristol-Myers Squibb, Celgene, Genentech, Regeneron, and Takeda, and consulting fees from Taiho Pharmaceuticals and Gilead not related to this study.

Simon Laban: Advisory Boards: Merck Sharp & Dohme (MSD), Bristol Myers Squibb (BMS), Astra Zeneca (AZ). Honoraria: MSD, BMS, AZ, Merck Serono.

Johannes Döscher: Advisory Boards: Merck Serono. Honoraria: Merck Serono.

Thomas K. Hoffmann: Advisory Boards: MSD, BMS. Honoraria: MSD, BMS, Merck Serono.

All other authors did not declare a conflict of interest.

## Ethical statement

The study received approval from the University Hospital of Bonn's internal review board (reference #174/13).

## CRediT authorship contribution statement

**Adrian v. Witzleben:** Writing – original draft, Visualization, Validation, Software, Formal analysis, Data curation. **Romain Remark:** Writing – review & editing, Data curation. **Christian Idel:** Writing – review & editing, Data curation, Conceptualization. **Julika Ribbat-Idel:** Writing – review & editing, Methodology, Data curation. **Rosemarie Krupar:** Writing – review & editing, Resources, Formal analysis, Data curation. **Andreas Schröck:** Writing – review & editing, Data curation. **Niklas Klümper:** Writing – review & editing, Investigation. **Johannes Doescher:** Writing – review & editing. **Andrew G. Sikora:** Writing – review & editing, Conceptualization. **Tsima Abou Kors:** Writing – review & editing. **Julius M Vahl:** Writing – review & editing. **Matthias Brand:** Writing – review & editing. **Michael Sonntag:** Writing – review & editing. **Cornelia Brunner:** Writing – review & editing. **Thomas K Hoffmann:** Writing – review & editing. **Sven Perner:** Writing – review & editing, Supervision, Methodology, Conceptualization. **Sacha Gnjatic:** Writing – review & editing, Supervision, Methodology, Investigation, Funding acquisition, Formal analysis, Data curation. **Simon Laban:** Writing – original draft, Visualization, Validation, Investigation, Funding acquisition, Formal analysis, Data curation, Conceptualization.

## Declaration of competing interest

Sacha Gnjatic reports research funding from Boehringer Ingelheim, Bristol-Myers Squibb, Celgene, Genentech, Regeneron, and Takeda, and consulting fees from Taiho Pharmaceuticals and Gilead not related to this study.

Simon Laban: Advisory Boards: Merck Sharp & Dohme (MSD), Bristol Myers Squibb (BMS), Astra Zeneca (AZ). Honoraria: MSD, BMS, AZ, Merck Serono.

Johannes Döscher: Advisory Boards: Merck Serono. Honoraria: Merck Serono.

Thomas K. Hoffmann: Advisory Boards: MSD, BMS. Honoraria: MSD, BMS, Merck Serono.

All other authors did not declare a conflict of interest.

## Appendix Supplementary materials

Image, application 1

Image, application 2
