# Supplementary material for: CD3 and PD-L1 tissue expression have synergistic value in head and neck squamous cell carcinoma prognosis
Source: Transl Oncol. 2026 Apr 16;68:102776. doi: 10.1016/j.tranon.2026.102776 (PMC13101706; doi:10.1016/j.tranon.2026.102776)
Supplement: Supplementary file 1 [file mmc1.docx]

**Supplementary Material**:

**Suppl. Figure 1:** Separated (by primary tumor region) and combined presentation of the CD3 infiltration with statistically different expression across the regions. *p*-value is presented.

**Suppl. Figure 2:** Example images of two cores of the TMA (tissue microarray) after staining with CD3. Low CD3 expression (left side), high CD3 expression (right side).

**Suppl. Figure 3: Overall survival (OS) of patients stratified by 3-way PD-L1 (CPS) expression.** OS (months) was significantly reduced for patients expressing with PD-L1 CPS <1, while PD-L1 CPS 1-19 and CPS ≥20 had the same superior survival. The *p*-values for statistical comparisons are indicated.

**Suppl. Figure 4: Overall survival (OS) and Recurrence-free survival (RFS) of patients stratified by combined CD3 and PD-L1 expression.** OS and RFS (months) was significantly reduced for patients expressing a high CD3 and a CPS ≥1. The *p*-values for comparisons are indicated.
