# Supplementary material for: CD3 and PD-L1 tissue expression have synergistic value in head and neck squamous cell carcinoma prognosis
Source: Transl Oncol. 2026 Apr 16;68:102776. doi: 10.1016/j.tranon.2026.102776 (PMC13101706; doi:10.1016/j.tranon.2026.102776)

Supplementary Figure 1

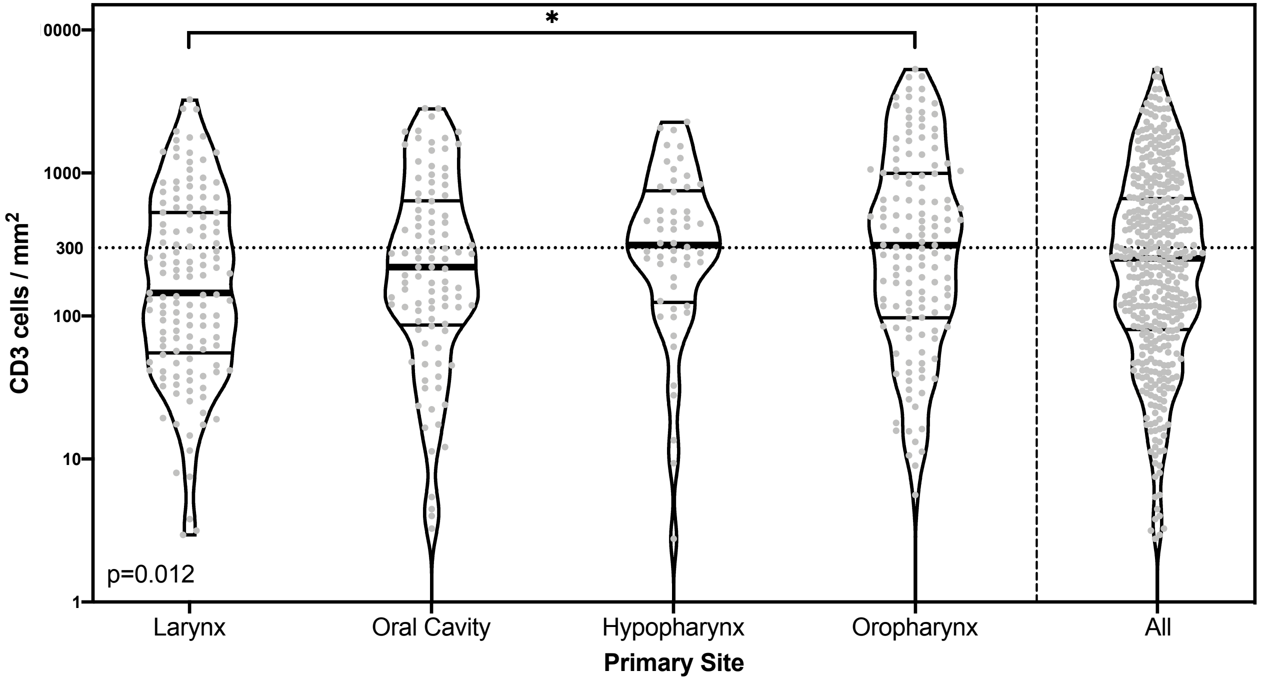

Supplementary Figure 2 – Example Image

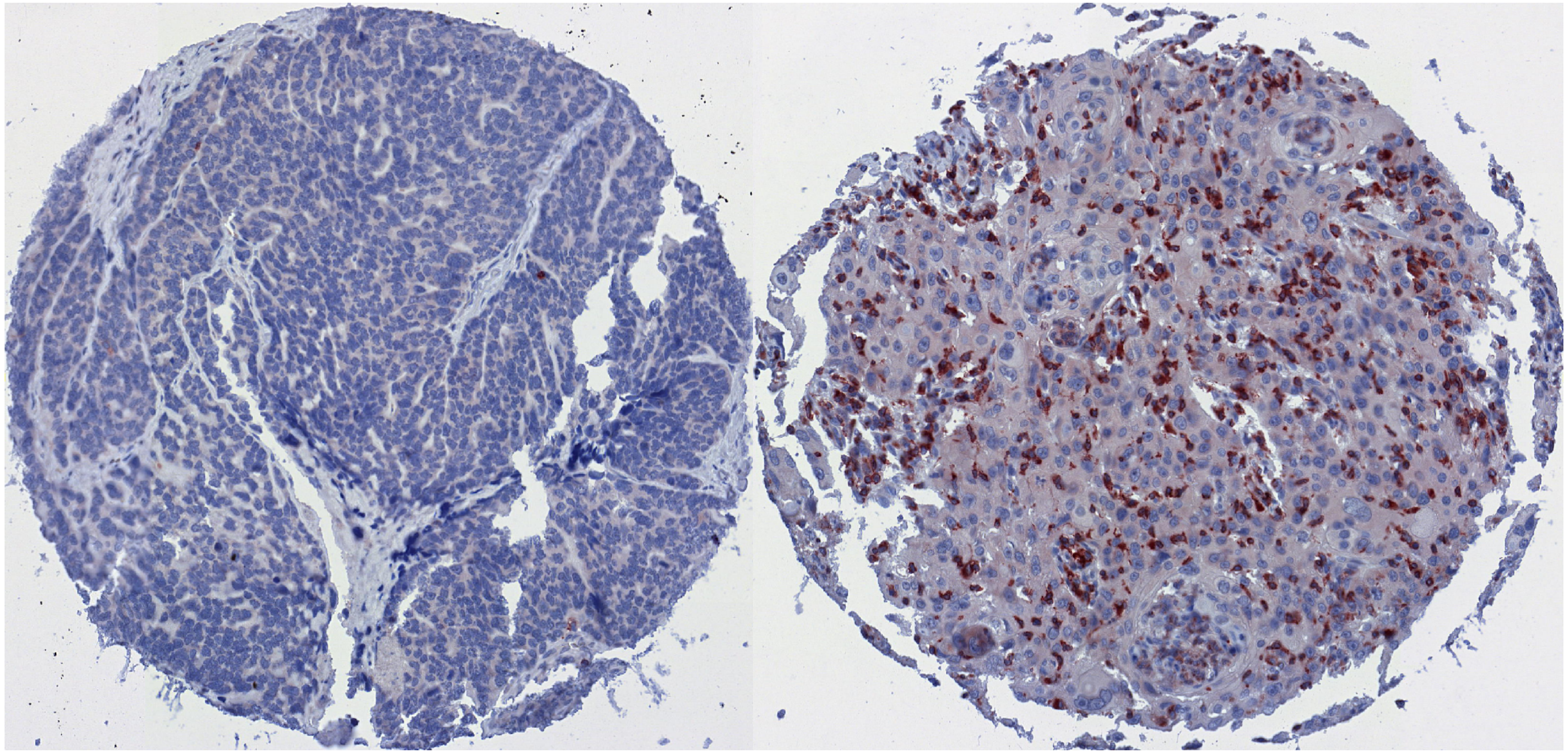

low CD3 high CD3

Supplementary Figure 3 – PDL1 Score 3-way grouped

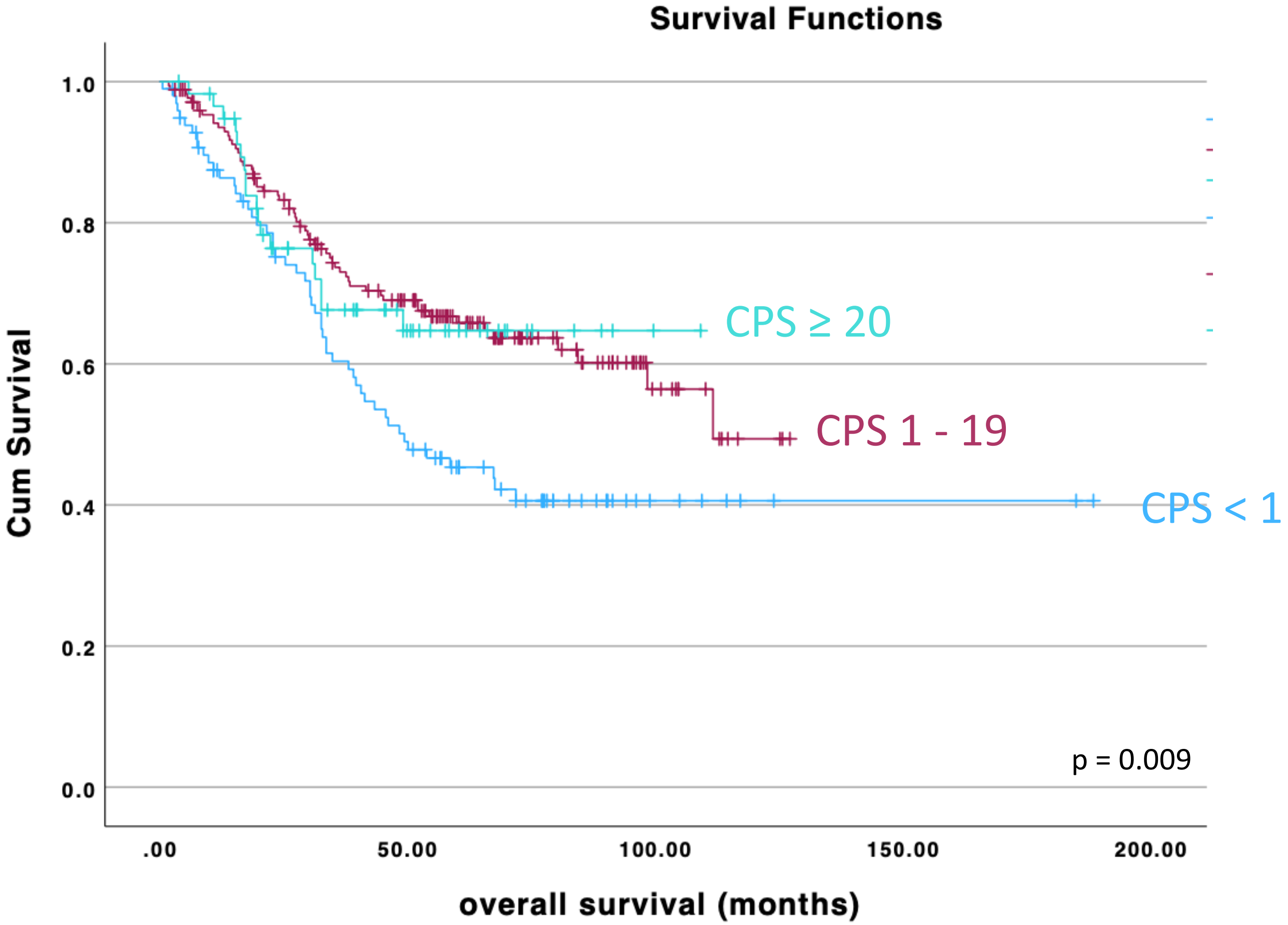

Supplementary Figure 4 – four groups combined

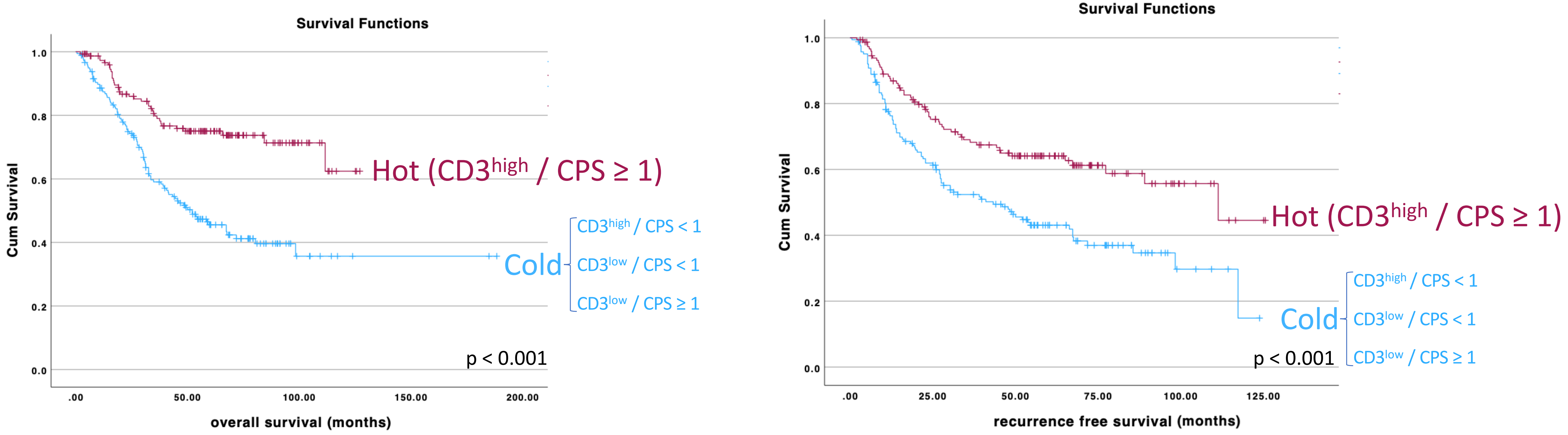

Supplement: Supplementary file 2 [file mmc2.pdf]
